# A Fast Beamforming Method to Localize an Acoustic Emission Source under Unknown Wave Speed

**DOI:** 10.3390/ma12050735

**Published:** 2019-03-04

**Authors:** Junfei Tai, Tian He, Qiang Pan, Dayi Zhang, Xiaoran Wang

**Affiliations:** 1 School of Transportation Science and Engineering, Beihang University, Beijing 100083, China; taijunfei@buaa.edu.cn (J.T.); hetian@buaa.edu.cn (T.H.); panqiang@buaa.edu.cn (Q.P.); wangxiaoran@buaa.edu.cn (X.W.); 2 School of Energy and Power Engineering, Beihang University, Beijing 100083, China

**Keywords:** acoustic emission, Bartlett beamforming, fast damage localization, structure health monitoring

## Abstract

The beamforming method is capable of localizing the acoustic emission source in a large-scale structure but its accuracy relies strongly on the assumed propagation speed and it is quite time consuming to apply in online monitoring. This paper proposes a fast beamforming method to localize an acoustic emission source in a thin-walled structure with unknown wave speed. Firstly, the Bartlett beamforming method (BBM) is introduced into broadband Lamb wave signal processing to develop an L-shape array-based damage source localization method for a thin-walled structure. Secondly, the fast Bartlett beamforming method (FBBM) is proposed based on the characteristics of BBM. Finally, the pencil-lead break test is carried out to validate the proposed method. The test results show that the FBBM can accurately localize the damage source by any given probable wave speed much more rapidly than traditional delay-and-sum beamforming.

## 1. Introduction

Ultrasonic techniques, including active and passive sensing, have been widely studied and applied in the field of structure health monitoring [1,2]. Passive ultrasonics, commonly referred to as acoustic emission (AE), has been extensively studied in real time damage monitoring. For example, Kundu et al. [3] proposed an AE source localization method based on Bayesian identification; they trained a probabilistic model to give probabilistic descriptors of damage locations in composites structures. Sikdar et al. [4,5] concentrated on the identification and monitoring problems of a damage-induced AE source in the advanced sandwich composite structure, and they proposed systematic methods based on an evolutionary algorithm and a localization algorithm to identify structure damage.

Damage localization based on AE technology is critical for damage detection and health evaluation, and this has led to a variety of applications [6]. Among many AE source localization technologies, the time difference of arrivals (TDOA) method has become the most widely studied and applied one in practical applications [7]. However, its inherent property makes the accuracy of the TDOA method highly sensitive to time difference and wave speed [8]. Therefore, many scholars concentrated on obtaining accurate time difference or accurate wave velocity. For instance, Ziola et al. [9] used the cross-correlation technique to study the characteristics of stress waves between special mode frequency and wave velocity. Hamstad et al. [10] determined the arrival time of signals by means of wavelet transform. Carpinteri [11] used the Akaike Information Criterion to recognize the arrival time of AE waves in concrete. In order to improve the localization accuracy, localization methods without wave speed have been studied. For example, Kundu et al. [12] studied the mathematical model based on the triangulation technique and developed a method without considering wave speed in the fields of damage localization for metal and composite materials [13]. Hensman et al. [14] proposed a time-reversal AE source localization method combined with statistical theory for structures with holes.

Although the acoustic emission localization methods have been extensively studied, there is still a great deal of work to be done [15]. The beamforming localization method is one of the promising attempts. McLaskey [16] and He et al. [17] introduced the beamforming method (BFM) to localize an AE source, which is easy to implement and exhibits high localization accuracy in a noisy environment [18]. Xiao et al. [19] found that the localization result of a uniform linear array of sensors is sensitive to wave speed only in the direction perpendicular to the linear array. Hence, they used two orthogonal linear arrays to determine the horizontal and vertical coordinates respectively and the results show that this is effective for source localization under inaccurate wave speed. Although BFM shows high localization precision and great robustness, its inherent drawback is its low computational efficiency. In other words, the more sensors used, the slower the calculation speed.

This paper aims to develop a fast and reliable localization method for AE sources on a thin-walled structure based on the Bartlett beamforming method (BBM) [20], no matter whether the wave speed is accurate or not. Since the method completely ignores the localization performance perpendicular to the array direction in exchange for consistency and accuracy of localizing effects parallel to the array direction, it can localize the AE source with high speed and precision by an L-shaped sensors array.

## 2. Principles

**Figure 1:**
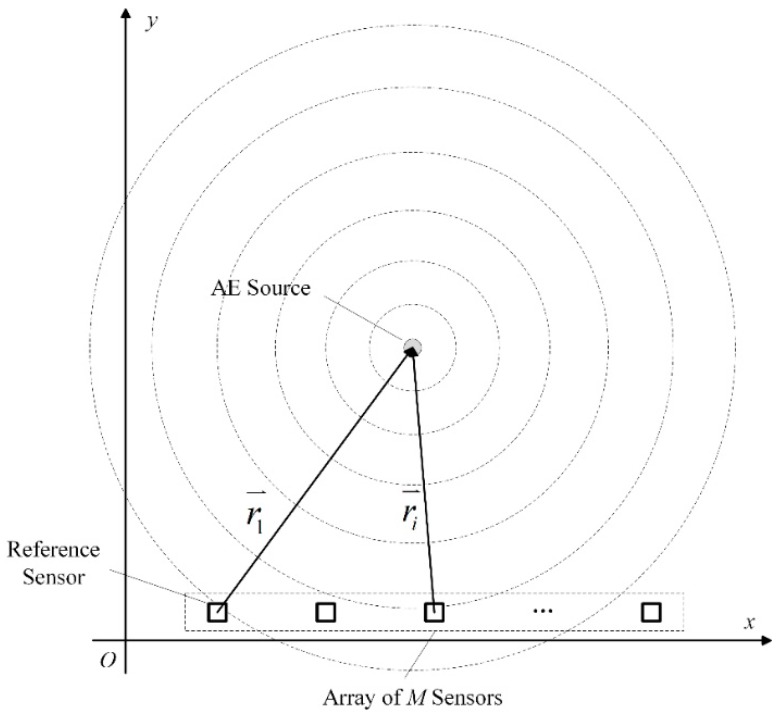
Schematic diagram of the near-field beamforming.

A frequency-domain weighted beamforming, named BBM, is introduced to establish the fast localization method. BBM, as a classic beamforming method, has been widely used to localize acoustic sources [20]. The schematic diagram of Bartlett beamforming in a plate-like structure is shown in Figure 1. For a two-dimensional plane in which *M* pieces of sensors are linearly arranged as shown in Figure 1, the signals $X\left(t\right)=[x_{1}\left(t\right),x_{2}\left(t\right),\cdots ,x_{M}\left(t\right){]}^{T}$ received by sensor array from the near-field AE source can be expressed by:

(1) X(t)=a(x,y,f)S(t)+N(t)

where N(t) is the noise received by *M* sensors of the array and can be given by $N\left(t\right)=[n_{1}\left(t\right),n_{2}\left(t\right),\cdots ,n_{M}\left(t\right){]}^{T}$. The function a(x,y,f) is defined as the steering vector, which represents an overall representation of the original signal S(t) transmitted from the position (x,y) under a particular array form:

(2) $$a\left(x,y,f\right)={\left[1,e^{-j2\pi f\tau_{2}},\cdots ,e^{-j2\pi f\tau_{M}}\right]}^{T}$$

where *f* is the center frequency of the signal, and $\tau_{i}$ is the time delay of the signal received by the *i*-th sensor relative to the reference signal:

(3) $$\tau_{i}=\frac{\left|\vec{r_{i}}\right|-\vec{\left|r_{1}\right|}}{c}$$

The signal represented by Equation (1) is processed by the weighting matrix $W^{H}\left(x,y,f\right)$. The dimension of $W^{H}\left(x,y,f\right)$ is 1×N, and the superscript *H* represents the conjugate transpose of the matrix W(x,y,f), which can be written by:

(4) $$W\left(x,y,f\right)={\left[W_{1}\left(x,y,f\right),W_{2}\left(x,y,f\right),\cdots ,W_{M}\left(x,y,f\right)\right]}^{T}$$

The element of W(x,y,f) may be a complex number whose modulo represents the weight on the amplitude of the output. The signal output after the weighting matrix processing is expressed by:

(5) $$Y\left(t\right)=W^{H}\left(x,y,f\right)X\left(t\right)$$

The signal processed by the weighting matrix is required to have the maximum power, and the mean square value of the signal is used to represent the average power of the signal:

(6) $$P=E\left[{\left|Y\left(t\right)\right|}^{2}\right]=W^{H}E\left[X\left(t\right)X^{H}\left(t\right)\right]W$$

where *E*[ ] represents the mean operator. Substituting Equation (1) into Equation (6), the power *P* can be rewritten by:

(7) $$P=E\left[{\left|S\left(t\right)\right|}^{2}\right]\left(W^{H}aa^{H}W\right)+\sigma^{2}{\left\|W\right\|}^{2}$$

where ‖·‖ represents the norm of vector, and $\sigma^{2}$ is the noise variance. When the noise variance is constant, a constraint must be established to ensure the signal to noise ratio of the output not be affected by the weighting matrix:

(8) ‖W‖=1

Under the constraint of Equation (8), Lagrange multiplier method is used to solve *W* to maximize the average signal power:

(9) $$W=\frac{a\left(x,y,f\right)}{\sqrt{a^{H}\left(x,y,f\right)a\left(x,y,f\right)}}$$

Substituting Equation (9) into Equation (6), the maximum power *P* can be reached:

(10) $$P=\frac{a^{H}\left(x,y,f\right)Ra\left(x,y,f\right)}{a^{H}\left(x,y,f\right)a\left(x,y,f\right)}$$

where $R=E\left[X\left(t\right)X^{H}\left(t\right)\right]$ is obtained by calculating the covariance matrix of the array signals. R has no relationship with the assumed wave speed, while it is affected by the propagation speed in the specific material. Because the change of propagation speed will affect the received signals X(t), and the change of X(t) is mainly about the relative phase position of signals from different sensor channels. That is to say, *R* still contains the information about where the true source is, and the function a(x,y,f) is used to find the most matched point (x,y). For the condition that the dimension of X(t) is M × T (where T represents the length of the digital signals), *R* will be a matrix with the dimension of M × M. The point (x,y) where the power *P* reaches maximum, is considered to be the acoustic source.

## 3. Implementation and Test of the Algorithm

### 3.1. Implementation and Improvement of the Proposed Algorithm

**Figure 2:**
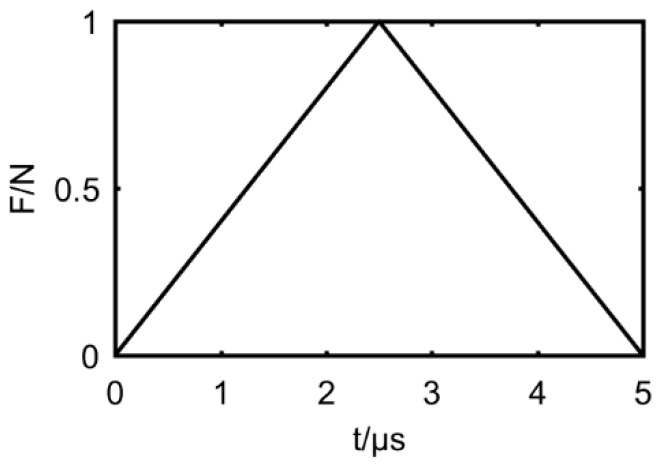
The time profile of transient triangular force.

The localization performance of the presented algorithm is tested by simulated signals of damage. The dimension of the thin-walled steel structure in the simulation is 700 × 700 × 5 mm. Density, elastic modulus, and the Poisson’s ratio of material are set as 7.8 × 10^3^ kg/m^3^, 2.09 × 10^11^ N/m^2^ and 0.3, respectively. The damage source was simulated by a pair of transient triangular forces lasting 5 μs, and the time profile of that is shown in Figure 2. Due to the short wavelength of the AE wave under normal conditions (for example, the bending wave is about 20 mm and the extending wave is about 35 mm in the AE wave with center frequency of 150 kHz), it is sufficient to mesh the geometry model by 1 × 1 mm^2^ to simulate the modes of the AE wave in the interested frequency band. The element type used in Abaqus (version 6.14, Dassault Systèmes Simulia Corp, Johnston, RI, USA) is C3D8R, which is a linearly reduced integration element with accurate displacement results and less computation time. Specific simulation details are described and validated in the literature [21].

**Figure 3:**
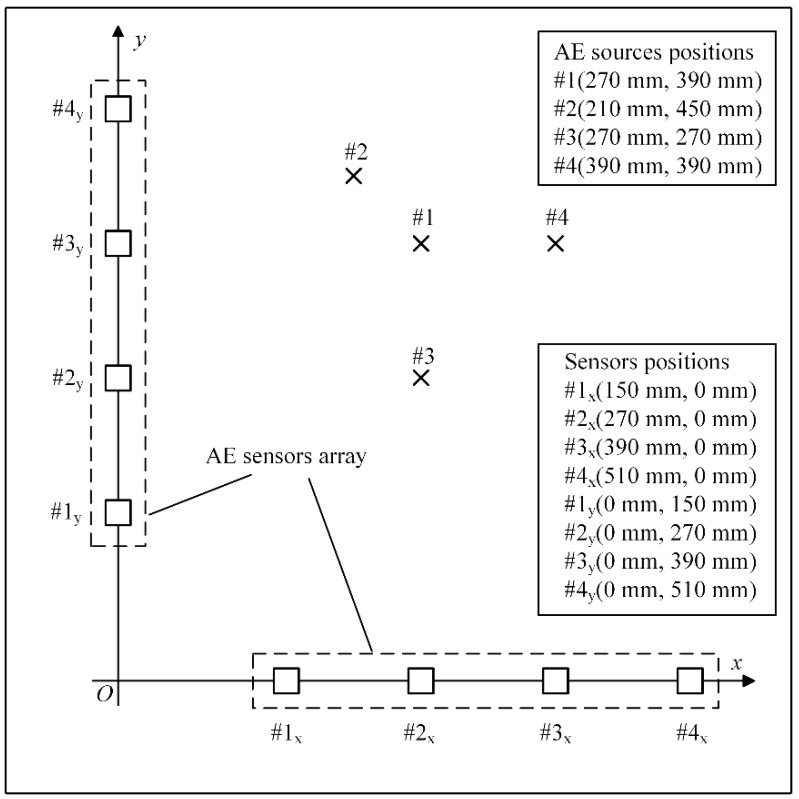
The arrangement of sensors and acoustic emission (AE) sources in the simulation.

Figure 3 shows the position of two perpendicular linear sensor arrays in a Cartesian coordinate system. The excitation positions of the damage sources at four different points are plotted in Figure 3.

**Figure 4:**
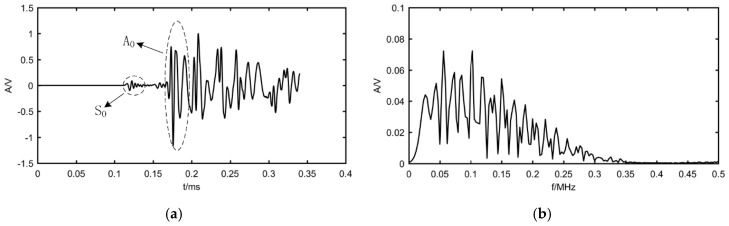
The AE signal acquired by simulation: (**a**) waveform of signal; (**b**) frequency spectrum.

Due to the dispersion and energy attenuation phenomena of the propagating AE wave, the excited transient triangular wave expands to a broadband signal through a complex propagation process. The time-domain waveform and frequency spectrum of the signal after propagating about 400 mm with a center frequency of 150 kHz are shown in Figure 4.

**Figure 5:**
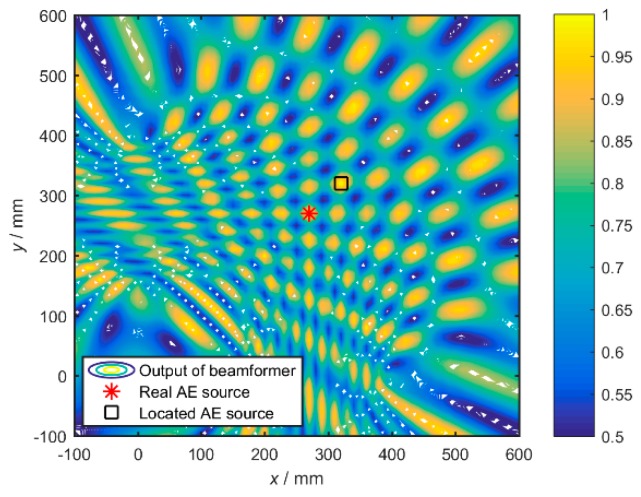
The preliminary localization determined by the *S*_0_ wave.

The localization performed by the *S*_0_ wave is shown in Figure 4a. The *S*_0_ wave is a symmetrical mode wave with low dispersion and good waveform consistency when it propagates far away from the source. The preliminary localization results of one set of data by using the proposed algorithm is shown in Figure 5.

As can be seen from Figure 4, the localization effect is not satisfying. It means that even if the dispersion of the S_0_ wave is not severe, the broadband AE signal cannot be used by Bartlett beamforming to localize sources effectively. Therefore, the solution method is presented by decomposing the broadband signal into several narrowband signals in the frequency domain, then using the Bartlett beamforming described in the second section to estimate the power spectrum on each sub-band, and finally summing them all to reach the overall result. The steering vector of the sub-band signal can be written as:

(11) $$a\left(x,y,f_{i}\right)={\left[1,e^{-j2\pi f_{i}\tau_{2}},\cdots ,e^{-j2\pi f_{i}\tau_{M}}\right]}^{T}$$

where *f*_i_ is the center frequency of the *i*-th sub-band. Therefore, the weighting matrix described by Equation (9) and the power *P* described by Equation (10) can be generalized by:

(12) $$W_{i}=\frac{a\left(x,y,f_{i}\right)}{\sqrt{a^{H}\left(x,y,f_{i}\right)a\left(x,y,f_{i}\right)}}$$

(13) $$P=\sum_{i=1}^{n}P_{i}=\sum_{i=1}^{n}{\frac{a^{H}\left(x,y,f_{i}\right)Ra\left(x,y,f_{i}\right)}{a^{H}\left(x,y,f_{i}\right)a\left(x,y,f_{i}\right)}}_{i}$$

where *n* is the number of sub-bands decomposed by the broadband signals.

**Figure 6:**
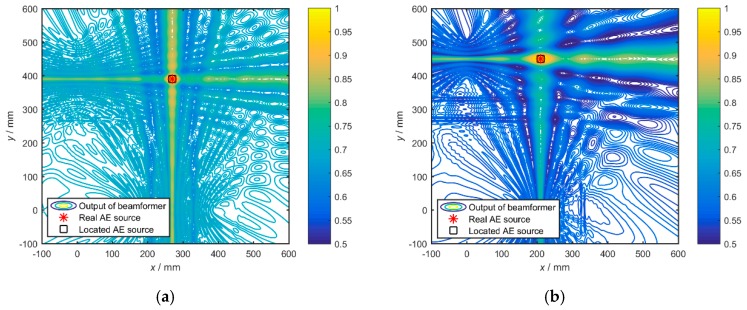

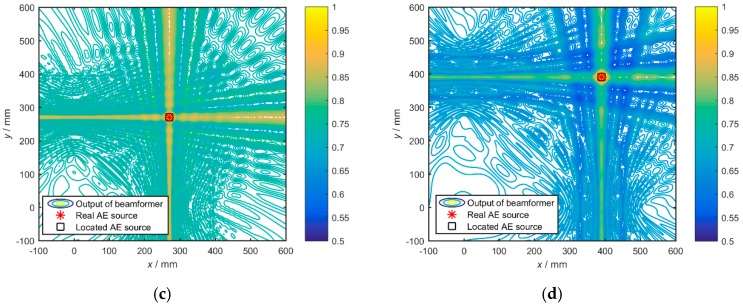
The localization results of four sets of AE sources: (**a**) Set 1; (**b**) Set 2; (**c**) Set 3; (**d**) Set 4.

The localizations result of four sets of signals is recalculated according to the improved algorithm and shown in Figure 6. The yellow lines represent the area with high calculated power *P*. It shows the case where the assumed propagation speed is 5300 m/s. Obviously, the localization results are quite accurate, and the improved broadband Bartlett beamforming has very good applicability in processing broadband AE signals.

In order to further evaluate the algorithm with inaccurate propagation speed, the assumed speed ranging from 2000 m/s to 12,000 m/s is used to localize with an interval of 2000 m/s. The results are summarized in Table 1. It can be seen that the proposed algorithm can accurately localize the sources even with inaccurate propagation speeds.

### 3.2. Principle of the Fast Algorithm

**Figure 7:**
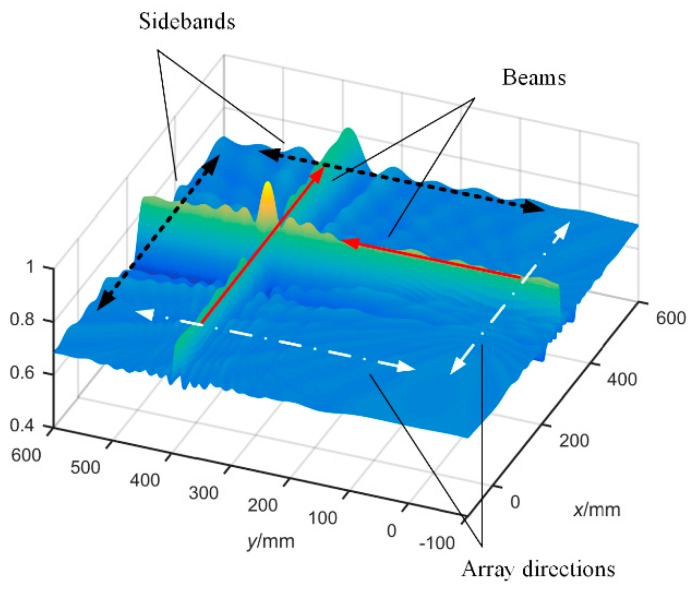
The three-dimension beamforming output diagram of a set of simulated signals.

**Figure 8:**
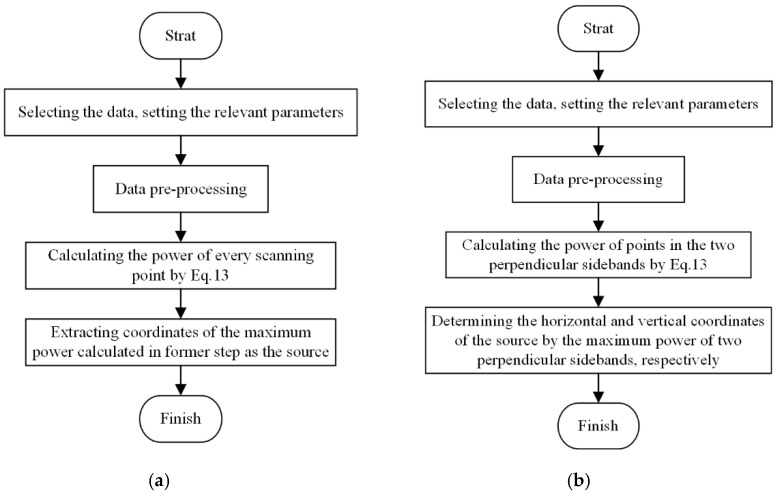
The flowcharts of the Bartlett beamforming method (BBM) and fast Bartlett beamforming method (FBBM): (**a**) flowchart of BBM; (**b**) flowchart of FBBM.

A three-dimensional localization diagram is shown in Figure 7, from which the beam characteristics of the proposed method can be observed. The array directions marked by white arrows are the location of linear sensor arrays. There are two beams perpendicular to the two arrays respectively, and the power of the entire beam is higher than the power of other regions. Therefore, one beam can clearly localize one coordinate of the AE source in the array direction, which means that the two perpendicular beams are able to determine the position of the AE source. However, there is no need to find out the complete two beams; the marginal profile (Sidebands) which is perpendicular to the beams is enough to localize the source position. It can greatly increase the localization efficiency. The flowcharts of BBM and FBBM are compared in Figure 8.

A comparison of Bartlett beamforming and fast Bartlett beamforming under the 1 mm scanning grid is conducted at propagation speed 5300 m/s. The average calculation times of two algorithms are measured by MATLAB in the same computer under the same condition. The results are shown in Table 2. It can be seen from Table 2 that the calculation speed of the fast Bartlett beamforming method (FBBM) is significantly higher than that of BBM while maintaining the same positioning accuracy.

The performance of fast Bartlett beamforming under different propagation speeds is calculated, and the results in Table 3 show that the fast Bartlett beamforming is as accurate as Bartlett beamforming under the propagation speeds ranging from 2000 m/s to 12,000 m/s.

## 4. Experimental Verification

**Figure 9:**
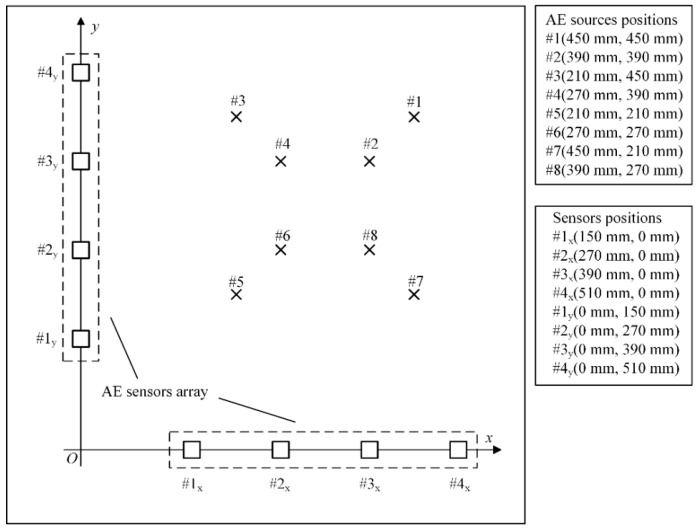
The arrangement of sensors and AE sources in the pencil-lead break (PLB) test.

**Figure 10:**
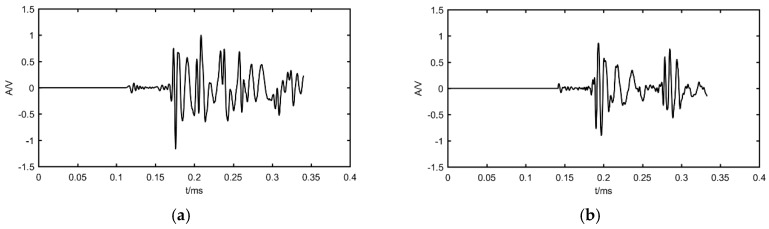
The comparison of AE signal acquired by simulation and experiment: (**a**) simulation signal; (**b**) experimental signal.

To further validate the algorithm performance, the experimental data in the literature by Xiao et al. [19] is used in this study and the result is compared with the localization method in that literature. Xiao et al. [19] used a pencil-lead break (PLB) test to generate a Hsu-Nielsen source and localized the sources by delay-and-sum beamforming. The PLB test is carried out on a homogeneous thin-walled steel plate with the size 700 × 700 × 5 mm. The layout of the L-shaped array and eight AE sources are shown in Figure 9. The sampling frequency is 3 MHz and the waveform of a set of experimental data compared with simulated data is shown in Figure 10. This indicates that the simulation signal has the main characteristic of the experimental AE signal.

Based on the collected data of 8 sources, the *S*_0_ waves in the signals were used for localization at different speeds, which is summarized in Table 4 in comparison with the paper [19]. It can be seen that the localization performance of fast Bartlett beamforming is generally better than that of delay-and-sum beamforming at the same propagation speed, and it can be applied to a wider range of propagation speeds with good stability. In addition, the calculation time of delay-and-sum beamforming and fast Bartlett beamforming under different scanning accuracy is compared. The calculation time is averaged by 8 sets of data at the same wave speed v = 5300 m/s, which is shown in Table 5. As can be seen from Table 5, the calculation of fast Bartlett beamforming under different scanning accuracy is much faster than delay-and-sum beamforming by about 50–420 times.

## 5. Conclusions

This paper proposes a fast and reliable algorithm to accurately localize the AE source based on an L-shaped array and Bartlett beamforming. The performance of the algorithm is analyzed by simulation and the PLB test. Some conclusions can be drawn as follows:

(1) Bartlett beamforming cannot be used directly for broadband lamb wave analysis. Based on the presented broadband processing method, the improved Bartlett beamforming has not only great localization performance, but also strong stability at different assumed propagation speeds.

(2) Based on the beam characteristics of Bartlett beamforming, this paper proposes a fast Bartlett beamforming method based on an L-shaped array. According to the simulation result, this method greatly improves the localization efficiency and maintains the accuracy of broadband Bartlett beamforming. Its calculation speed is 224 times faster than that of Bartlett beamforming under the same conditions.

(3) According to the experimental results, the fast Bartlett beamforming method can obtain accurate localization results with a wide range of propagation speed, and its calculation speeds under different scanning grids are 50–420 times faster than delay-and-sum beamforming.

(4) The fast Bartlett beamforming method proposed in this paper has accurate and fast localization performance in a wide range of inaccurate assumed propagation speeds. It is suitable for monitoring and localizing damage sources in large plate structures.

## Figures and Tables

**Table 1:** Localization results of Bartlett beamforming under inaccurate wave speed.

Speed (m·s^−1^)	Location							
	AE Sources (mm, mm)							
	Set 1		Set 2		Set 3		Set 4	
	*x*	*y*	*x*	*y*	*x*	*y*	*x*	*y*
	270	390	210	450	270	270	390	390
2000	270	390	210	450	270	270	390	390
4000	270	390	210	450	270	270	390	390
6000	270	390	210	450	270	270	390	390
8000	270	390	210	450	270	270	390	390
10,000	270	390	210	450	270	270	390	390
12,000	270	390	210	450	270	270	390	390

**Table 2:** Localization comparison of the BBM and the FBBM at v = 5300 m/s.

Results	AE Sources (mm, mm)								Average Time (s)
	Set 1		Set 2		Set 3		Set 4		
	*x*	*y*	*x*	*y*	*x*	*y*	*x*	*y*	
	270	390	210	450	270	270	390	270	
BBM	270	390	210	450	270	270	390	270	224.38
FBBM	270	390	210	450	270	270	390	270	0.72

**Table 3:** Localization results of fast Bartlett beamforming under inaccurate wave speed.

Speed (m·s^−1^)	Location							
	AE Sources (mm, mm)							
	Set 1		Set 2		Set 3		Set 4	
	*x*	*y*	*x*	*y*	*x*	*y*	*x*	*y*
	270	390	210	450	270	270	390	390
2000	270	390	210	450	270	270	390	390
4000	270	390	210	450	270	270	390	390
6000	270	390	210	450	270	270	390	390
8000	270	390	210	450	270	270	390	390
10,000	270	390	210	450	270	270	390	390
12,000	270	390	210	450	270	270	390	390

**Table 4:** Localization results comparison by PLB test.

**Categories of Algorithm**	**Propagation Speed (m·s^−1^)**	**AE Sources (mm, mm)**							
		**Set 1**		**Set 2**		**Set 3**		**Set 4**	
		***x***	***y***	***x***	***y***	***x***	***y***	***x***	***y***
		**450**	**450**	**390**	**390**	**210**	**450**	**270**	**390**
Delay-and-sum beamforming [19]	2000	440	450	390	390	210	460	260	390
	2500	450	460	390	390	200	460	260	390
	3000	450	460	390	390	200	470	260	390
	3500	450	460	390	420	200	470	260	390
	4000	450	460	390	420	200	470	260	390
Fast Bartlett beamforming	2000	450	450	390	390	210	450	270	390
	2500	450	450	390	390	210	450	270	390
	3000	450	450	390	390	210	450	270	390
	3500	450	450	390	390	210	450	270	390
	4000	450	450	390	390	210	450	270	390
	6000	450	450	390	390	210	450	270	390
	8000	450	450	390	390	210	450	270	390
	10,000	450	450	389	390	210	450	270	390
	12,000	450	449	390	390	211	449	270	390
**Categories of Algorithm**	**Propagation Speed (m·s^−1^)**	**Set 5**		**Set 6**		**Set 7**		**Set 8**	
		***x***	***y***	***x***	***y***	***x***	***x***	***x***	***y***
		**210**	**210**	**270**	**270**	**450**	**210**	**390**	**270**
Delay-and-sum beamforming [19]	2000	210	250	270	260	470	260	390	290
	2500	210	240	270	260	470	230	390	260
	3000	210	240	260	260	460	220	390	260
	3500	210	250	260	260	460	220	390	260
	4000	210	250	260	260	460	220	390	270
Fast Bartlett beamforming	2000	210	210	270	270	450	210	390	270
	2500	210	210	270	270	450	210	390	270
	3000	210	210	270	270	450	210	390	270
	3500	210	210	270	270	450	210	390	270
	4000	210	210	270	270	450	210	390	270
	6000	210	210	270	270	450	210	390	270
	8000	210	211	270	270	450	210	390	270
	10,000	210	208	270	270	450	210	390	270
	12,000	210	213	270	270	450	210	390	270

**Table 5:** The average calculation time of two kinds of algorithm with different scanning accuracy.

Scanning Accuracy (mm)	Delay-and-Sum Beamforming (s)	Fast Bartlett Beamforming (s)	Ratio
5	8.49	0.17	50.69
2	49.16	0.35	139.96
1	193.26	0.72	269.49
0.5	780.19	2.13	366.50
0.2	4887.19	11.63	420.09
